# Susceptibility profile of clinical and food-associated *Listeria monocytogenes* strains to a commercial phage product using different test methods

**DOI:** 10.3389/fmicb.2025.1614697

**Published:** 2025-08-11

**Authors:** Christoph Brieske, Christina Böhnlein, Hui Zhi Low, Charles M. A. P. Franz

**Affiliations:** Department of Microbiology and Biotechnology, Max Rubner-Institut, Federal Research Institute of Nutrition and Food, Kiel, Germany

**Keywords:** *Listeria monocytogenes*, phage P100, phage-susceptibility, spot assay, plaque assay, flow cytometry, optical density, colony reduction

## Abstract

Foodborne bacterial pathogens continue to pose a significant global health and economic burden, with *Listeria monocytogenes* being a persistent risk due to its frequent involvement in outbreaks and food recalls. Bacteriophage-based products are promising tools for enhancing food safety, yet systematic evaluations across genetically diverse *L. monocytogenes* strains are limited. In this study, we assessed the efficacy of a commercially available *Listeria*-specific phage product against 50 whole-genome-sequenced clinical and food-associated *L. monocytogenes* isolates recently collected in Germany. Traditional spot and plaque assays indicated 70–76% susceptibility, whereas viability-based methods, including colony reduction, OD_600_ measurement, and flow cytometry, demonstrated substantial bacterial reduction across all isolates within 24 h. Notably, flow cytometry revealed a marked decline in viable cells as early as 3 h post-treatment. By systematically comparing susceptibility assays, we argue that modern viability-based methods assessing microbial load reduction offer key advantages over classical plaque assays for evaluating phage efficacy in food safety applications. While plaque assays remain valuable primarily for determining infectivity, reduction-based approaches have the potential to serve as a measure of antimicrobial performance in biocontrol settings.

## 1 Introduction

Foodborne bacterial pathogens have a significantly negative impact on health and the economy worldwide. In 2023, 35 foodborne outbreaks in the EU were linked to *Listeria* (*L.*) *monocytogenes*, with 133 cases of illness, of which 11 ended fatally (European Food Safety Authority, 2024). *Listeria monocytogenes* is a Gram-positive, rod-shaped pathogen that infects animals and humans (Murray et al., 1926; Schlech et al., 1983). Due to its cold resistance (Tasara and Stephan, 2006), *L. monocytogenes* can grow and multiply at refrigeration temperatures, enabling the pathogen to easily persist and multiply in foods despite the efficiency of modern cold chains. In ready-to-eat (RTE) foods, contamination with *L. monocytogenes* can be found in fish and fish products, products of meat origin, vegetables, juices, cheeses, and other products (European Food Safety Authority, 2021). After ingestion of contaminated food, *L. monocytogenes* may cause listeriosis. Clinically, a mild infection manifests as gastroenteritis, but can also lead to severe, invasive diseases such as sepsis, meningitis, and meningoencephalitis. Pregnancy-associated infections can manifest as miscarriage or neonatal sepsis (Koopmans et al., 2023).

Bacteriophage (phage)-based products claiming to have broad host ranges against *L. monocytogenes* have been commercially available for several years. Although these products are already approved for use in countries such as Israel, Canada, China, Switzerland, Australia, and New Zealand (Abd-El Wahab et al., 2023; Costa et al., 2023), they are still awaiting approval in Germany. A phage used to combat *L. monocytogenes* in foods is phage P100, which is commercially available as the Phageguard L preparation. This lytic phage is fully characterized, and there are a few studies on the efficacy of this phage (Carlton et al., 2005; Guenther et al., 2009; Soni et al., 2010; European Food Safety Authority, 2016; Gutiérrez et al., 2017). For application of phages in foods, a point of concern is whether the phages are capable of killing all, or at least the majority, of the relevant circulating strains of the pathogens in question. An effective phage product used for improving food safety should obviously have the broadest susceptibility range possible.

Studies that demonstrate the efficacy of this phage in foodstuffs often only focused on very limited isolates (Carlton et al., 2005; Guenther et al., 2009; Soni et al., 2010; Gutiérrez et al., 2017). Fister et al. (2016) screened 486 *L. monocytogenes* isolates obtained from 59 dairies over 15 years. However, these isolates were not genotyped, and thus the study could not account for potential multiple isolates of the same strain. In addition, the range was narrow since the isolates were only obtained from dairy plants in a limited geographic region. As far as we know, no study has yet investigated the susceptibility profile of recently isolated, whole genome-sequenced and genotypically distinct clinical and food-associated *L. monocytogenes* strains to the commercial P100 phage preparation. The clinical isolates used in this study offered a unique advantage, as they represented highly prevalent sequence types (STs) of recent listeriosis cases in Germany, as was determined by multi-locus sequence typing (Halbedel et al., 2024).

To test phage susceptibility of bacterial strains, the spot and plaque assays are considered the gold standard. However, these techniques are time-consuming, and the evaluation of plaques and spots is subject to interpersonal variability and subjectivity. Recently, alternative methods have been developed to overcome these disadvantages. The innovations lie in automation, which reduces analysis time and resource utilization, and/or the use of new and more sophisticated detection techniques, e.g., flow cytometry, that make it possible to move from qualitative “endpoint” results (visual observation with the naked eye) to quantitative measurements (Daubie et al., 2022). Flow cytometry is a well-established method for analyzing eukaryotic cells in biomedical research, and in recent years, the method has also found increased application in microbiological studies (Verthé and Verstraete, 2006; Low et al., 2020; Egido et al., 2023). Flow cytometry is an accurate, sensitive, and powerful technique. With an appropriate combination of fluorescent dyes, flow cytometry allows visualization of physiological differences between cells, such as discrimination between live and dead bacterial cells (Ambriz-Aviña et al., 2014; Fernández-Fernández et al., 2022).

As there are a number of different methods available for testing phage susceptibility, it is also important to know whether there are any differences in terms of suitability between those methods. The aim of this study, therefore, was to systematically investigate the susceptibility range of clinical (*n* = 20 most common genotypes) and food-associated (*n* = 30) *L. monocytogenes* strains to a P100 phage-based product. The strains were recently isolated in Germany, whole-genome sequenced, and genotypically distinct (Halbedel et al., 2018; Halbedel et al., 2019; Lüth et al., 2020; Halbedel et al., 2021; Halbedel et al., 2024). In addition, the study also aimed to compare different methods for determining phage susceptibility and to evaluate their strengths and weaknesses.

## 2 Methods

### 2.1 Microorganisms and growth conditions

A total of 20 clinical and 30 food-associated *L. monocytogenes* strains were analyzed for phage susceptibility in this study. The strains are listed in Tables 1, 2. *Listeria innocua* (*L. innocua*) WSLC 2627 was used as a reference strain for phage titer verification. The commercial phage product Phageguard L (formerly Listex; Micreos Food Safety B. V., Wageningen, The Netherlands) targeting *L. monocytogenes* was used. Phageguard L contains phage P100 at a concentration of 2 × 10^11^ plaque-forming units (PFU)/mL, as indicated by the manufacturer.

**Table 1:** Food-associated *Listeria monocytogenes* isolates (*n* = 30).

Isolate no.	Origin	Sero group	Sequence type (ST)	Clonal complex	Cluster type	Reference
19-LI01144-0	Brunswick sausage	IIb	3	CC3	16,348	N/A
19-LI01376-0	Bauernknacker (smoked sausage)	IIa	121	CC121	13,307	N/A
19-LI01463-0	Smoked Salmon ham, raw	IVb	2	CC2	13,329	N/A
19-LI01475-0	Teewurst	IIc	9	CC9	13,308	N/A
20-LI00049-0	Bratwurst, smoked	IVb	2	CC2	13,747	N/A
20-LI00052-0	Bratwurst, smoked	IIa	155	CC155	13,773	N/A
20-LI00069-0	Mettwurst, finely minced	IVb	1	CC1	3,672	N/A
20-LI00075-0	Bauernbratwurst	IIa	37	CC37	13,894	N/A
20-LI00218-0	Mackerel fillet, smoked	IIa	121	CC121	13,900	N/A
20-LI00373-0	Raw sausages, firm to cut	IIa	91	CC14	14,086	N/A
20-LI00412-0	Landjäger	IIa	16	CC8	7,906	N/A
20-LI00440-0	Fish, smoked	IVb	1	CC1	4,522	N/A
20-LI00441-0	Salmon, smoked	IVb	6	CC6	4,160	N/A
20-LI00449-0	Onion sausage	IVb	1	CC1	6,529	N/A
20-LI00563-0	Salmon, smoked	IIa	37	CC37	7,559	Lachmann et al. (2022)
20-LI00702-0	Salmon, smoked	IIa	155	CC155	1,128	Lachmann et al. (2022)
20-LI00734-1	Cooked ham, cured	IVb	6	CC6	4,465	N/A
20-LI00847-0	Fish, smoked	IIa	155	CC155	8,452	N/A
20-LI01696-0	Salmon, smoked	IIb	3	CC3	14,754	N/A
20-LI01813-0	Salmon, smoked	IIa	101	CC101	8,245	N/A
21-LI00451-0	Hot-smoked salmon	IIa	121	CC121	2,808	N/A
21-LI00472-0	Salmon, smoked	IIc	9	CC9	5,470	N/A
21-LI00474-0	Salmon, smoked	IIb	87	CC87	1,138	Lachmann et al. (2022)
21-LI00480-0	Cooked ham, cured	IIb	3	CC3	15,093	N/A
21-LI01023-0	Mackerel fillet, smoked	IIa	8	CC8	15,549	N/A
21-LI01731-0	Salmon ham, raw, smoked	IIa	451	CC11	4,584	N/A
21-LI01764-0	Bacon, raw, smoked	IIa	7	CC7	15,975	N/A
21-LI01787-0	Smoked Greenland halibut	IVb	6	CC6	3,163	N/A
22-LI00090-0	Salmon, smoked	IIa	37	CC37	5,488	Lachmann et al. (2022)
22-LI00719-0	Trout fillet, smoked	IIc	9	CC9	4,619	N/A

**Table 2:** Clinical *L. monocytogenes* isolates (*n* = 20, most prevalent sequence types).

Isolate no.	Cluster	Serogroup	Sequence type (ST)	Frequency of STs in % (Halbedel et al., 2024)	Cluster type	Reference
14–03633	Beta1	IIb	5	2	2,789	Lüth et al. (2020)
15–01121	Eta1	IVb	2	5	1,114	Halbedel et al. (2021) and Halbedel et al. (2019)
16–00461	Xi2	IIa	451	4	3,997	Halbedel et al. (2018)
16–01823	Chi1	IIa	14	1	2,966	Halbedel et al. (2018)
16–02860	Zeta1	IIa	403	1	40	Halbedel et al. (2018)
16–05164	Epsilon2	IIa	7	2	4,348	Halbedel et al. (2018)
17–01398	Rho3	IIc	9	1	1,690	Lachmann et al. (2022)
17–01914	My2	IIa	173	3	3,242	Lachmann et al. (2021)
17–02546	Eta3	IIa	26	1	4,045	Fischer et al. (2020)
17–05075	Omikron1	IIa	155	4	1,128	Lachmann et al. (2022)
18–00325	Ypsilon3	IIa	121	1	5,554	Halbedel et al. (2024)
18–00792	Tau4	IVb	4	4	6,460	Halbedel et al. (2024)
18–00833	My3	IIb	224	1	4,466	Halbedel et al. (2024)
18–04415	Sigma1	IIa	8	12	2,521	Fischer et al. (2021) and Lachmann et al. (2021)
18–04540	Epsilon1a	IVb	6	17	4,465	Halbedel et al. (2021) and Halbedel et al. (2020)
18–04580	Pi5	IIb	3	2	6,665	Halbedel et al. (2024)
19–00661	Kappa3	IIb	87	1	4,557	Halbedel et al. (2024)
19–02942	Theta3a	IVb	249	1	4,449	Halbedel et al. (2024)
19–03903	Eta5	IIa	37	4	5,488	Halbedel et al. (2024)
20–06258	Kappa8	IVb	1	15	4,961	Halbedel et al. (2024)

All bacteria strains were routinely cultured in Brain Heart Infusion (BHI) broth and on BHI agar (Carl Roth, Karlsruhe, Germany). For long-term storage, strains were grown for 18 hours at 20°C (food-associated isolates) or 37°C (clinical isolates, to reflect the human host environment) and then stored at −80°C in BHI broth supplemented with 20% glycerol.

For propagation, strains were streaked onto BHI agar plates and incubated for 2 days at 20°C, after which the plates were stored at 4°C. For experimental use, single colonies were inoculated into BHI broth and cultured overnight (19 ± 1 hours) at 20°C. All experiments were conducted at 20°C to simulate environmental conditions relevant to food processing settings, where *L. monocytogenes* contamination and phage application, e.g., via surface spraying, are likely to occur.

### 2.2 Non-host and phage-negative controls

Three types of controls were included: (i) a non-host control strain, (ii) no-phage controls, and (iii) controls with heat-inactivated phages. The Gram-positive bacterium *Lactococcus lactis* NZ9000 was used as the non-host control strain. No-phage controls consisted of samples without phage addition, while additional controls were treated with heat-inactivated phages. Phage inactivation was achieved by heating the phage preparation at 80°C for 15 minutes, followed by storage at 4°C. Complete inactivation was confirmed using spot assays and flow cytometry.

### 2.3 Spot and plaque assays

For spot tests, 100 μL bacterial overnight culture was mixed with 3 mL BHI soft agar (0.7% w/v agar) and poured onto BHI agar plates according to Kutter (2009). After solidification of the agar, 5 μL of each dilution of a tenfold serial phage dilution in SM buffer (0.05 M Tris–HCl pH 7.5, 0.58% w/v NaCl, 0.2% w/v MgSO_4_·7H_2_O), ranging in concentrations from 1.6 × 10^10^ to 1.6 × 10^3^ PFU/mL, were spotted onto the agar surface (~8 × 10^7^ to 8 phages/spot and ~2.8 × 10^6^ cells/spot corresponds to a multiplicity of infection (MOI) of ~29 to 2.9 × 10^−6^). A 5 μL volume of SM buffer without phage served as a control. For heat-inactivated phage control, 5 μL of heat-inactivated phage product was spotted. The plates were incubated at 20°C for 24–48 h. Spots were then evaluated for lysis.

For double-agar overlay plaque assays, 100 μL overnight culture and 10 μL phage dilution of the abovementioned 10-fold serial phage dilutions (1.6 × 10^10^ to 1.6 × 10^3^ phages/mL and ~2 × 10^9^ cells/mL corresponds to a MOI of ~0.8 to 8 × 10^−8^) were mixed and preincubated at RT for 5 min, then mixed with 3 mL BHI soft agar and poured onto BHI agar plates based on the methodology described by Kropinski et al. (2009). As negative controls, the SM buffer without phage or heat-inactivated phages was added. The plates were incubated at 20°C for 24–48 h. Plaque numbers were counted, and PFU/mL was calculated. For titer validation of the phage product, *Listeria innocua* (*L. innocua*) WSLC 2627 was used as the host bacterium, which is also used by the company for titer determination. The experiment was performed in duplicate with three technical replicates each.

### 2.4 Colony reduction assay

Bacteria overnight cultures were diluted 1:10^7^ with SM buffer, and 100 μL of this dilution was plated onto BHI agar using the spread plate method. After absorption, 200 μL 1% v/v phage product dilution in SM buffer was plated onto BHI agar (3.2 × 10^8^ phages/agar plate and ~2 × 10^2^ cells/agar plate corresponds to an MOI of ~1.6 × 10^6^). As control, the SM buffer without phage or heat-inactivated phage was used. *Lactococcus* (*L*.) *lactis* NZ9000 was used instead of *L. monocytogenes* as a bacterial non-host negative control. The plates were incubated at 20°C for approximately 48 h, and colonies were counted to determine the number of viable bacteria. The experiment was performed in duplicate with three technical replicates each.

### 2.5 Optical density 600 nm (OD_600_) measurement

*Listeria monocytogenes* strains were propagated in BHI broth with and without phage product at 20°C to determine bacterial cell number reduction using optical density measurement. Bacterial overnight cultures were diluted 1:2000 with BHI broth (corresponds to approx. 1 × 10^6^ cells/mL), and each strain was incubated with 1% v/v phage product (1.6 × 10^9^ phages/mL and ~1 × 10^6^ cells/mL corresponds to a MOI of ~1,600) at a final volume of 100 μL in a well of a 96 well plate at 20°C. As a negative control, no phage was added. For heat-inactivated phage control, 1% v/v heat-inactivated phage product was used. *L. lactis* served as a bacterial non-host negative control. After 24 h of incubation, the absorbance was determined at 600 nm using a TECAN Spark 10 M microplate reader (TECAN, Männedorf, Switzerland). The experiment was performed in triplicate.

### 2.6 Flow cytometric live/dead lysis assay

Live/dead bacterial cell analysis was performed by flow cytometry to determine the viable bacteria count (on the basis of membrane intactness), as well as to quantify phage lysis of host bacteria, similar to that described by Low et al. (2020). Live/dead staining solution was freshly prepared by diluting SYTO 13 and 20 mM propidium iodide (PI) (Thermo Fisher Scientific, Waltham, Massachusetts) 1:4,000 and 1:8,000 in 0.25 × Ringer’s solution, respectively. For quantification of phage lysis, overnight cultures of bacteria were diluted 1:2,000 into BHI broth (corresponds to approx. 1 × 10^6^ cells/mL) and incubated with 1% v/v phage product (1.6 × 10^9^ phages/mL and 1 × 10^6^ cells/mL corresponds to a MOI of ~1,600), final volume 400 μL/well, at 20°C using deepwell plates (Nerbe, Winsen/Luhe, Germany). As a negative control, no phage was added. For heat-inactivated phage control, 1% v/v heat-inactivated phage product was used. *L. lactis* served as a further bacterial non-host negative control. After 0, 3, and 24 h of incubation, a live/dead analysis was performed by diluting the bacterial suspension 1:50 into live/dead staining solution in 96 well plates and incubating for 30 min at room temperature before data acquisition on the CytoFLEX flow cytometer (Beckman Coulter, Krefeld, Germany) by measuring the number of events in 10 μL of sample. Bacterial cells were gated by their forward and sideward scatter, and then analyzed for their SYTO 13 and PI fluorescence. Flow cytometric viable cell counts of a few strains were validated using the spread-plate method. For this, 100 μL of a 1:10 serial dilution series of bacteria and bacteria + phage in 0.25 × Ringer’s solution were plated onto BHI agar. After overnight incubation at 37°C, colonies were counted to determine the number of viable bacteria per milliliter. The experiments were performed in triplicate.

### 2.7 Software

Flow cytometric data were analyzed using CytExpert version 2.5 software (Beckman Coulter, Krefeld, Germany). For statistical analysis, two-tailed t-tests were performed, and graphs were generated using Microsoft Excel and SigmaPlot, version 14.0 (Systat Software Inc., San Jose, California). *p*-values below 0.05 were considered statistically significant.

## 3 Results

### 3.1 Spot and plaque assays

**Figure 1:**
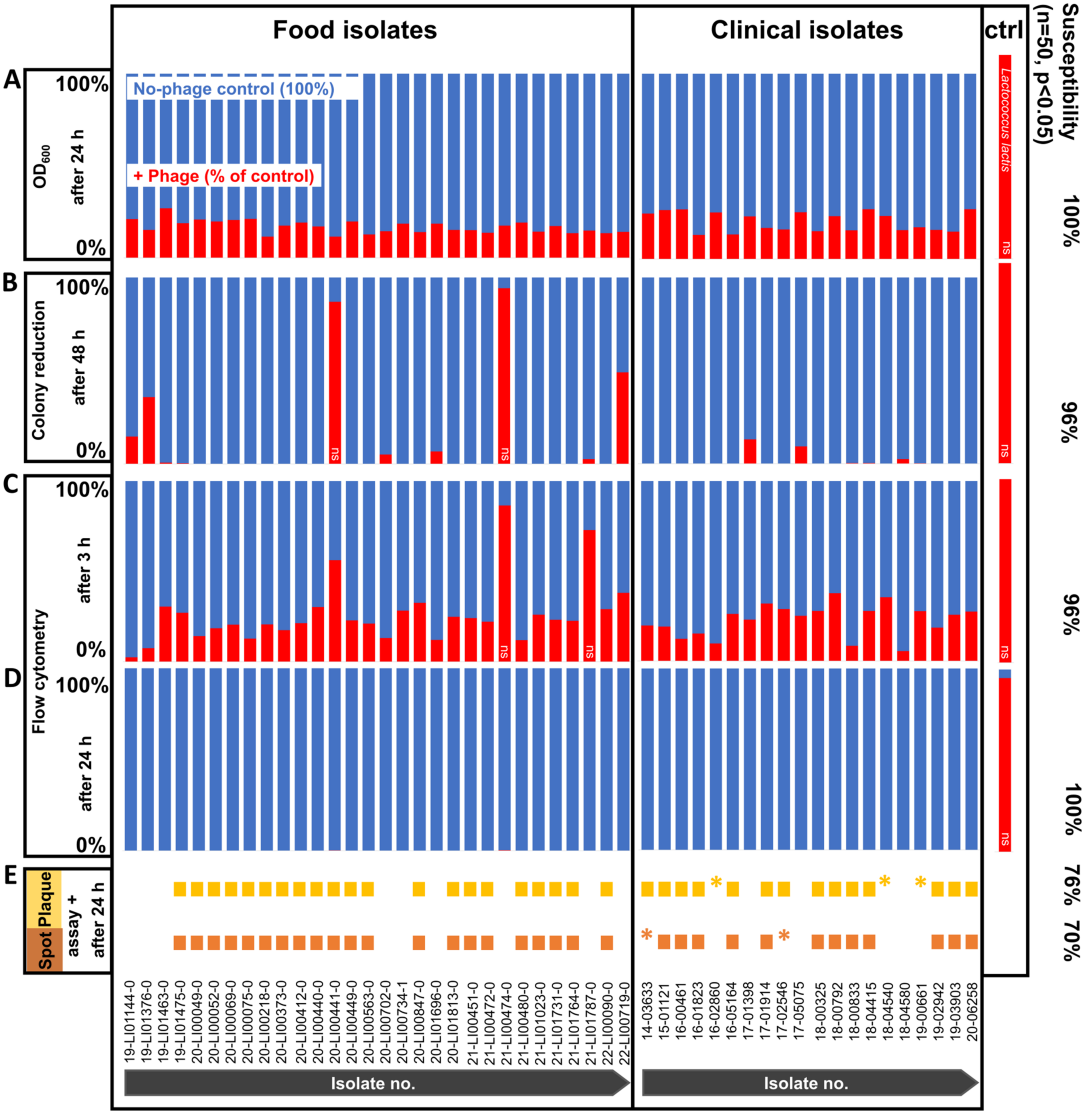
Phage susceptibility testing of *Listeria monocytogenes* strains (*n* = 50) and non-host control (ctrl) *Lactococcus lactis* at 20°C with and without phage treatment. **(A)** OD_600_ measurement after 24 h. **(B)** Colony reduction after 48 h. **(C)** Flow cytometry after 3 h. **(D)** Flow cytometry after 24 h. **(E)** Spot and plaque assays after 24 h. All values are expressed as percentages, with each no-phage control (blue bars) set to 100%. Cell reductions are calculated as a percentage of control (red bars). The data are shown as the mean values of the results from three biological replicates in one experiment (OD_600_ measurement, flow cytometry) or three technical replicates from two experiments each (colony reduction). Two-tailed Student’s *t*-test was performed on the replicates for each isolate to test for statistical significance between treatment with phage versus no-phage control. All isolates were statistically significantly reduced (*p* < 0.05) with the exception of those marked with ns, ns not significant (*p* > 0.05), *very small plaques.

Of the 50 *L. monocytogenes* isolates, 70 and 76% of the strains were found to be susceptible to the commercial phage product in spot and plaque assays, respectively (Figure 1E). This was visually determined by observing the formation of clear plaques or spots. Although plaques could be visually detected, no plaque counts could be determined for strains 14–03633 and 17–02546, or strains 16–02860, 18–04540, and 19–00661 in the spot and plaque assays, respectively, as these were too small to be visually discerned and counted and prolonged incubation did also not enhance the plaque size or clarity. No lysis could be observed in the remaining 30 and 24% of the isolates, respectively. PFU counts of spot and plaque assays are listed in Supplementary Tables S1, S2.

### 3.2 Colony reduction assay

When using the colony reduction method, phage incubation reduced *L. monocytogenes* cell counts slightly to substantially (6–100%) after 48 h for all of the isolates compared to the negative control without phage. However, the counts of 2 of the isolates (20-LI00441-0, 21-LI00474-0) showed a weak reduction, which was too low to be statistically significant (Figure 1B). The *L. monocytogenes* counts per agar plate of no-phage control vs. phage-treated *L. monocytogenes* are shown in Supplementary Tables S1, S2.

### 3.3 Optical density measurement

Of the 50 *L*. *monocytogenes* isolates, all (100%) strains were found to be susceptible to the phage product after 24 h incubation at 20°C using the OD_600_ measurement and were statistically significant reduced (Figure 1A). These susceptible isolates showed no turbidity development after phage incubation due to lack of growth and lysis of the bacterial cells. The OD_600_ values of the isolates incubated with phage product and no-phage controls are listed in Supplementary Tables S1, S2.

### 3.4 Flow cytometric live/dead lysis assay

**Figure 2:**
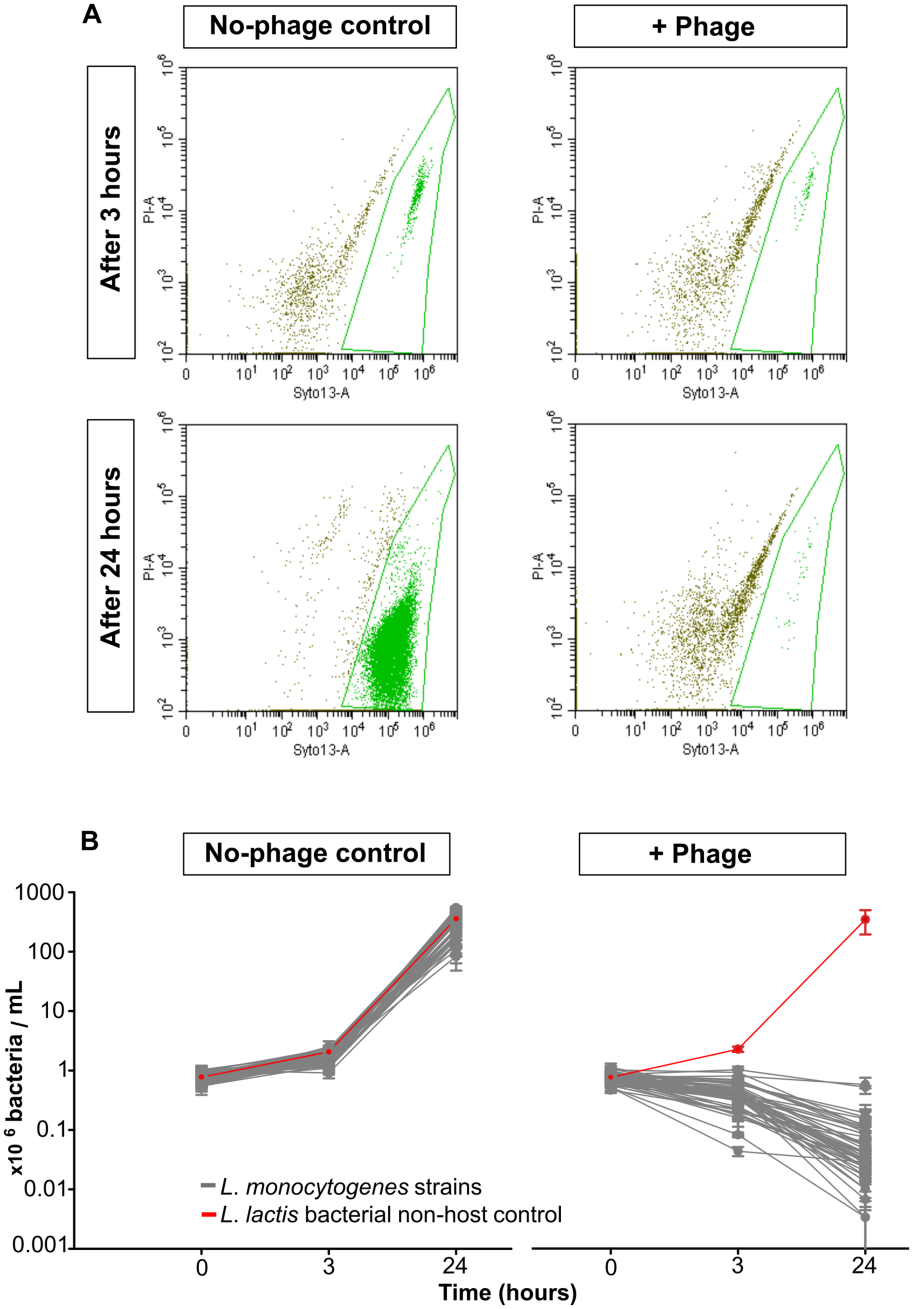
Flow cytometric live/dead staining of *L*. *monocytogenes* strains and controls at 20°C with and without phage treatment. **(A)** Flow cytometric dot plots of *L. monocytogenes* strain 20-LI00069-0, untreated control (left) and phage-treated (right) after 3 h (upper) and 24 h (lower). **(B)** Live cell reduction of 50 *L. monocytogenes* isolates and non-host negative control *L. lactis* after 0, 3, and 24 h (right). No-phage controls (left). The data are shown as the mean values and standard deviations of the results from three biological replicates in one experiment.

**Figure 3:**
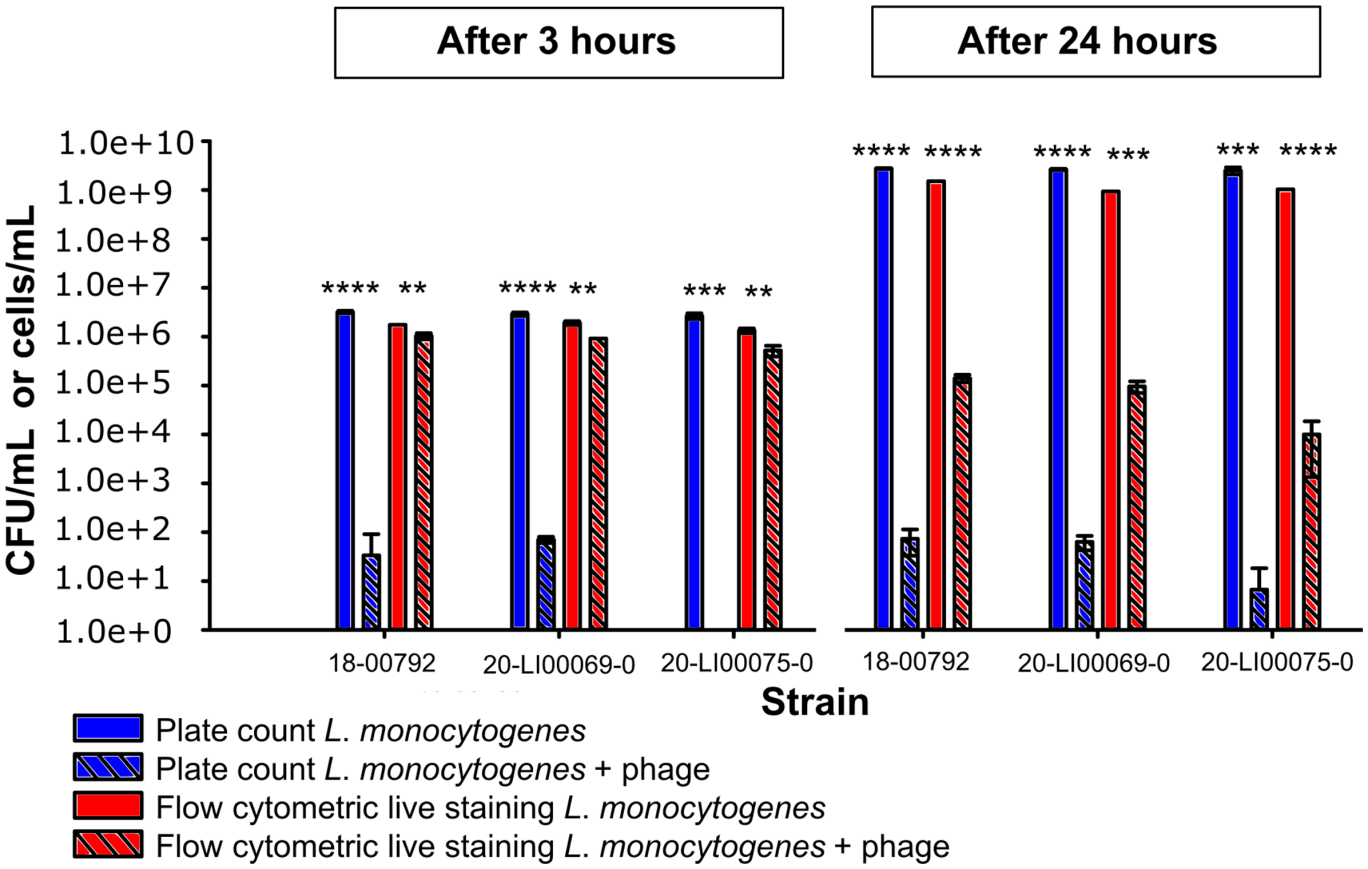
Comparison of *L*. *monocytogenes* viable cell numbers (cells/mL) obtained by flow cytometric live staining and by the spread plate method (CFU/mL) after 3 h and 24 h incubation at 20°C with phage and without phage. The data are shown as the mean values and standard deviations of the results from three biological replicates in one experiment. Statistically significant bacterial cell reduction is marked with asterisks (**p < 0.01, ***p < 0.001, ****p < 0.0001).

With flow cytometric live/dead staining, a substantial reduction in live cell count with the phage product (9–98%) compared to the controls without phages could be observed for assays with all 50 strains, even after only 3 h of phage incubation. A total of 96% of the strains were statistically significant reduced, as 2 isolates (21-LI00474-0, 21-LI01787-0) were too weakly reduced to be statistically significant (Figure 1C). An increased, statistically significant reduction of 99.7 to 100% could be achieved for assays with all 50 strains after 24 h incubation (Figure 1D). The flow cytometric dot plots and cell counts of the isolates incubated with phage and no-phage controls over time are shown in Figure 2, Supplementary Figure S1, and Supplementary Tables S1, S2. The spread-plate method of enumerating bacteria without phage showed comparable cell counts to the live cell counts obtained by flow cytometry (approx. 10^6^ cells/mL or CFU/mL after 3 h and 10^9^ cells/mL or CFU/mL after 24 h, shown in Figure 3). In contrast to this, the flow cytometric live cell counts of bacteria incubated with phage were higher than those of bacteria enumerated from plates (Figure 3).

## 4 Discussion

The Phageguard L phage product tested in this study showed a very broad spectrum of activity against both clinical and food-associated *L. monocytogenes* isolates that have been recently isolated in Germany. All of the 50 strains tested were phage-susceptible in at least two of the 5 susceptibility testing methods performed.

The host range describes the spectrum of bacteria that a phage can successfully and productively infect when obligately lytic phages produce offspring and lyse the host (de Jonge et al., 2019). In our study, we focused on susceptibility and bacterial cell reduction instead of host range, because a reduction in the bacterial pathogen count is the primary objective for food safety applications, whether through productive infection or lysis from without. A total of 70 and 76% of our strains tested susceptible to the phage in spot and plaque assays, respectively. The plaques occurred in a variety of sizes, depending on the *L. monocytogenes* strain used. Plaque formation failed to occur in 24–30% of the strains tested, even though all of those strains were very clearly demonstrated to be susceptible to the phage when the other test methods, such as colony reduction, OD_600_ measurement, and flow cytometry, were used.

The classical, plaque-based cultural methods continue to represent the gold standard for phage susceptibility testing (Fong et al., 2021; Maffei et al., 2021; Cunningham et al., 2022; Daubie et al., 2022). They are based on bacterial lysis within a soft agar, which is visually detected by zones of clearing. Theoretically, one plaque is formed by one infectious phage particle, which proliferates in the soft agar (Abedon and Yin, 2009). These tests, therefore, primarily allow a quantitative determination of the phage titer and characterization of individual plaque morphology. By definition, a bacterial strain is susceptible when a plaque or a zone of lysis is formed. Here, it is important to reflect upon whether the parameter that the plaque-based methods measure, i.e., the formation of a zone of lysis, is actually the best indicator of susceptibility. Furthermore, 24 h and in some cases, 48 h incubation time at 20°C were necessary for plaque formation. Plaque formation might also fail on the technicality of a soft agar layer that is too hard (0.7% agar). Micreos, which distributes this phage product, uses 0.3% soft agar in plaque-based assays. With regard to the occurrence of pathogens in the food, the initial bacterial concentration that was used experimentally in the soft agar may be considered to be extremely high (~10^7^CFU/mL). This results in low MOIs that do not reflect the low *L. monocytogenes* titers and thus are not reflective of contamination conditions that would normally occur in a food processing plant (European Food Safety Authority, 2013). They also do not simulate the intended use of the phage product, which effectively treats the phage preparation as a decontamination product that “floods” any contaminating *Listeria* with high phage titers.

On the other hand, the colony reduction method mimicked conditions that might more closely reflect the phage preparation in actual *in situ* use. The low bacteria count and high MOI employed were more representative of low *L. monocytogenes* counts that could be expected to be found in contaminated foods and usage conditions of the phage product. Susceptibility is then clearly indicated by the restriction of the growth of the bacterial colonies, or lack thereof. Nevertheless, the colony reduction method was highly labor-intensive and required 48 h for the colonies to reach a sufficient size for proper counting.

The OD_600_ and flow cytometric methods gave susceptibility profiles that corresponded well to the colony reduction method. While the OD_600_ method targets bacterial culture turbidity as an indicator of growth, the flow cytometric live/dead method directly and rapidly measures the actual live bacteria count based on cell membrane integrity. It is important to note, however, that membrane integrity alone does not fully capture bacterial viability, and the use of additional markers, such as indicators of metabolic activity, can provide a more comprehensive assessment (Carvalho et al., 2024). Flow cytometric live/dead staining allowed the detection of live cell count reductions for all 50 strains tested, even after only 3 h with phage incubation. Bacterial counts obtained via the spread-plate method without phage treatment closely matched flow cytometric live/dead staining results, confirming the accuracy of the flow cytometry approach for assessing viable cells. However, for bacteria incubated with phage, flow cytometry reported considerably higher viable cell counts than the plate method. The reason for this lies in the methodology. Flow cytometry allows real-time measurement of viable cells, whereas the spread-plate method requires intact, uninfected cells that can multiply and form colonies and does not account for cells infected after incubation with phages. In some cases, only a limited reduction of culturable cells was observed after 24 h (Figure 3), which may suggest early regrowth of less susceptible subpopulations. Although we did not specifically investigate the development of non-susceptible phenotypes within this study, we cannot fully exclude this possibility. This is also supported by the observation that only a certain percentage of OD_600_ is suppressed by the presence of phages (Figure 1A), potentially indicating survival or regrowth of a fraction of the bacterial population. However, plaque assays performed for all tested strains indicated that the majority of strains were initially susceptible to phage P100, and in additional longer-term experiments (data not shown), resistant colonies were only observed after several days. This suggests that the emergence of resistant genotypes likely played a minor role within the 24-h timeframe used in this study.

The commonality uniting all the colony reduction, OD_600,_ and flow cytometric methods is that different unlike the spot and plaque assays, these are direct and indirect quantitative methods of bacterial reduction resulting from the phage preparation. The chosen range of MOIs in spot and plaque assays reflects standard conditions commonly used in phage susceptibility testing and was selected to ensure comparability with previous studies. For the methods measuring bacteria reduction, a minimum bacterial cell count had to be used to ensure accurate detection and reproducibility of the measurement, which in turn resulted in a higher MOI that was variable depending on the experimental setup.

This study also investigated whether the observed differences in phage susceptibility could be linked to the genetic background of the tested strains, including serogroups and STs. However, no consistent correlation was identified. Only two strains showed a clearly reduced inhibition in the colony reduction assay, but it remains unclear whether this is due to specific mutations. Addressing this would require detailed comparative genomic analyses across a larger set of strains, which is beyond the scope of the current study. The two strains that showed only a slight, non-statistically significant reduction in the colony reduction assay are highly relevant for challenge assays and phage effectiveness testing, which is usually conducted with very few strains of *L. monocytogenes* that are already known to be susceptible. There is, therefore, a need to also perform such tests on strains that are less susceptible, for stress testing the phage preparation.

While we have defined susceptibility as a statistically significant reduction of bacteria in the presence of the phage product, it might be prudent in the future to determine a more suitable cutoff point for susceptibility. A more suitable cutoff point for deciding whether a bacterial strain is susceptible could be set, for example, by a comparison with the actual strength of the reduction in food matrices, and is subject to further study.

In view of the growing problem of antibiotic resistance, we observe that our experiences are also relevant to the rising therapeutic use of phages. Rapid screening for suitable phages can be decisive for the success of phage treatment in patients. Current plaque-based methods, such as spot and plaque assays, require sufficient time for the bacteria to grow confluently, typically within 16–24 h (Moelling et al., 2018; Aslam et al., 2020), and, as we have shown, give very different susceptibility profiles in comparison to reduction-based approaches.

When establishing susceptibility testing methods, it is paramount to consider the most relevant methodology for the intended purpose. Among the phage susceptibility test methods used in this investigation, the colony reduction assay most closely reflects the practical application of phages on foodstuffs for pathogen biocontrol. Flow cytometry proved to be the fastest, delivering highly reproducible data within 3 h, and the results are comparable to those of the colony reduction assay.

In conclusion, our results demonstrate the effectiveness of the *Listeria* phage-based product Phageguard L on a larger number of genotyped clinical and food-associated *L. monocytogenes* strains in liquid broth and semi-solid medium, emphasizing its potential for the reduction of listeriosis. A limitation of the study is the uncertainty of how the results translate to the food matrix setting, in which many intrinsic factors may affect phage efficiency. However, results obtained under optimal *in vitro* culture conditions reveal the potential of the phage preparation in affecting a wide spectrum of currently circulating food and clinical *L. monocytogenes* isolates. The study also showed that it may be prudent to rethink the use of plaque and spot assays, which are currently considered to be the gold standard for phage susceptibility testing, in favor of methods that directly address bacterial reduction, e.g., colony counting, flow cytometry, and OD_600_ measurement. While plaque assays remain valuable for determining infectivity, reduction-based approaches have the potential to serve as a measure of antimicrobial performance in biocontrol settings. Finally, while the link between phage susceptibility and strain genotypes was explored, no consistent correlation could be identified, in part due to the very small number of strains that showed reduced susceptibility.

## Data Availability

The original contributions presented in the study are included in the article/Supplementary material, further inquiries can be directed to the corresponding author.

## Supplementary material

SUPPLEMENTARY FIGURE S1 Dot plots of flow cytometric live/dead staining of non-host negative control *L. lactis* at 20°C after 3 and 24 h with (right) and without (left) phage treatment. Green dots represent live-stained bacteria cells.

SUPPLEMENTARY TABLE S1 Raw data of clinical and food-associated *L. monocytogenes* isolates used for phage susceptibility testing.

SUPPLEMENTARY TABLE S2 Calculated data of clinical and food-associated *L. monocytogenes* isolates used for phage susceptibility testing.

SUPPLEMENTARY TABLE S3 Raw data of *L. monocytogenes* live cell numbers obtained by flow cytometric live staining and by the spread-plate method after 3 and 24 h incubation at 20°C with phage and without phage.
