# Supplementary figures and images for: Susceptibility profile of clinical and food-associated Listeria monocytogenes strains to a commercial phage product using different test methods
Source: Front Microbiol. 2025 Aug 11;16:1614697. doi: 10.3389/fmicb.2025.1614697 (PMC12382164; doi:10.3389/fmicb.2025.1614697)

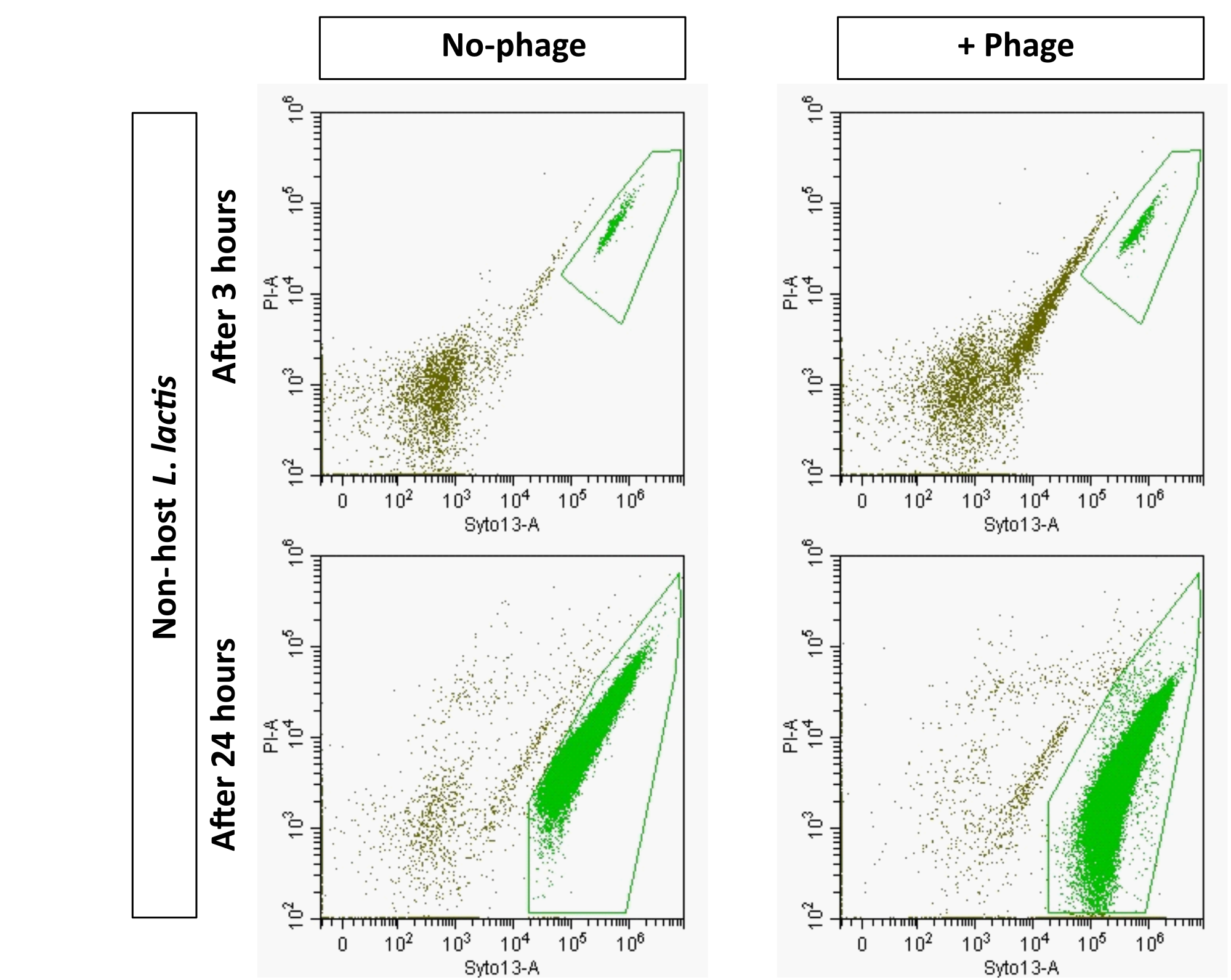

Supplement: SUPPLEMENTARY FIGURE S1 — Dot plots of flow cytometric live/dead staining of non-host negative control L. lactis at 20°C after 3 and 24 h with (right) and without (left) phage treatment. Green dots represent live-stained bacteria cells. [file Image_1.tiff]
